# Long-term myocardial deformation abnormalities and biomarker associations in pediatric myopericarditis: a speckle-tracking echocardiographic study

**DOI:** 10.3389/fcvm.2026.1938858

**Published:** 2026-10-28

**Authors:** Sule Arici, Selen Karagozlu, Elif Erolu Kibris, Figen Akalın

**Affiliations:** 1 Department of Pediatric Cardiology, Kosuyolu Yuksek Ihtisas Egitim ve Arastirma Hastanesi, Istanbul, Türkiye; 2 Department of Pediatric Cardiology, Marmara Universitesi Tip Fakultesi, Istanbul, Türkiye; 3 Department of Pediatric Cardiology, Ankara Etlik Entegre Saglik Kampusu, Ankara, Türkiye

**Keywords:** myopericarditis, echocardiography, speckle tracking, ventricular dysfunction, left, myocardium, inflammation mediators

## Abstract

**Background and objectives:**

Subclinical myocardial deformation abnormalities may be detectable during long-term follow-up in pediatric patients with a history of myopericarditis despite apparently normal conventional echocardiographic findings. This study aimed to comprehensively evaluate long-term myocardial deformation abnormalities using speckle-tracking echocardiography and to assess their associations with acute-phase inflammatory and cardiac biomarkers.

**Methods:**

Thirty pediatric patients with a history of myopericarditis and 22 age- and sex-matched healthy controls were included. Acute-phase clinical, laboratory, electrocardiographic, and echocardiographic data were retrospectively collected. At long-term follow-up, comprehensive echocardiographic evaluation including tissue Doppler imaging and speckle-tracking–derived longitudinal and circumferential strain analysis was prospectively performed. Associations between acute-phase biomarkers and long-term echocardiographic findings were analyzed.

**Results:**

At a mean follow-up of 32.8 ± 12.0 months, patients demonstrated significantly reduced global longitudinal strain (−12.43 ± 6.67% vs. −17.77 ± 1.94%, *p* < 0.001) and global circumferential strain (−16.04 ± 12.50% vs. −25.14 ± 4.73%, *p* = 0.002) compared with controls despite largely preserved conventional systolic function. Segmental longitudinal and circumferential strain parameters were also impaired, accompanied by increased time-to-peak strain dispersion. Higher neutrophil-to-lymphocyte ratio and systemic immune-inflammation index values correlated with less negative global longitudinal strain values, while CRP correlated with impaired global circumferential strain.

**Conclusions:**

Pediatric patients with a history of myopericarditis may exhibit subclinical myocardial deformation abnormalities at long-term follow-up despite apparently preserved conventional echocardiographic findings. Speckle-tracking echocardiography may provide incremental value for detecting subtle myocardial deformation abnormalities beyond conventional systolic assessment. Exploratory associations were observed between acute-phase inflammatory biomarkers and long-term myocardial deformation parameters; however, larger prospective studies are needed to clarify whether these findings have causal or clinical significance.

## Introduction

Myopericarditis is an inflammatory disease characterized by the concomitant involvement of the myocardium and pericardium, typically presenting with chest pain, electrocardiographic abnormalities, and elevated cardiac biomarkers (1). Although the clinical course in pediatric patients is generally considered more favorable than that of classic myocarditis, the disease spectrum may vary from mild self-limited forms to clinically significant myocardial involvement (1, 2). In children and adolescents, myopericarditis is increasingly recognized as an important cause of acute chest pain and elevated troponin levels, frequently leading to hospitalization and advanced cardiac evaluation (3). Despite generally preserved conventional systolic function and favorable short-term outcomes, concerns remain regarding subclinical myocardial abnormalities during long-term follow-up (2, 4).

Conventional echocardiographic parameters such as ejection fraction and fractional shortening may remain within normal limits despite ongoing myocardial injury or subtle impairment in myocardial mechanics. Therefore, advanced echocardiographic techniques, particularly two-dimensional speckle-tracking echocardiography (STE), have gained increasing attention for the detection of subclinical myocardial abnormalities. STE enables quantitative assessment of myocardial deformation through the analysis of longitudinal and circumferential strain parameters, providing greater sensitivity for the detection of early myocardial abnormalities compared with conventional echocardiography (5, 6). Previous studies have demonstrated the utility of strain imaging in identifying subtle myocardial abnormalities in various pediatric and inflammatory cardiac conditions, even in the presence of preserved conventional systolic function (7–10).

Although strain echocardiography has been increasingly used in pediatric myocarditis and other inflammatory cardiac conditions, data regarding long-term myocardial mechanics in pediatric myopericarditis remain limited. In particular, studies providing a comprehensive assessment of both longitudinal and circumferential strain parameters together with time-to-peak deformation indices in children with a history of myopericarditis are scarce (6, 10–12). Furthermore, the relationship between acute-phase inflammatory and cardiac biomarkers and long-term myocardial deformation findings has not been well characterized in pediatric myopericarditis. We hypothesized that pediatric patients with a history of myopericarditis would demonstrate abnormalities in myocardial deformation parameters during long-term follow-up compared with healthy controls, and that acute-phase inflammatory and cardiac biomarker levels would be associated with long-term myocardial deformation parameters. Therefore, the present study aimed to evaluate long-term conventional and advanced echocardiographic findings, including tissue Doppler imaging and speckle-tracking–derived myocardial deformation parameters, in pediatric patients with a history of myopericarditis, and to investigate their associations with acute-phase clinical and laboratory markers.

## Materials and methods

### Study design and patient population

This study was designed as a hybrid observational study incorporating both retrospective and prospective components. The diagnosis had been established by the treating pediatric cardiology team based on the overall clinical presentation, characterized by sharp chest pain compatible with pericardial inflammation, elevated cardiac troponin T levels indicating myocardial injury, and generally preserved left ventricular systolic function on conventional echocardiography. Electrocardiographic changes and associated inflammatory or infectious symptoms were considered supportive findings when present. Inclusion criteria were age <18 years, an established diagnosis of isolated myopericarditis by the pediatric cardiology team, availability of acute-phase clinical and laboratory data, and completion of long-term echocardiographic follow-up including speckle-tracking analysis. Patients with concomitant structural or other significant cardiac disease were excluded.

During the acute episode, all patients were managed with bed rest and received ibuprofen at a dose of 30–45 mg/kg/day in three divided doses, together with proton pump inhibitor therapy for gastroprotection. Competitive sports and strenuous physical activity were restricted for 6 months. In patients presenting with fever and elevated C-reactive protein levels, empirical intravenous ampicillin–sulbactam therapy was additionally administered.

A total of 30 pediatric patients were included. Clinical, laboratory, electrocardiographic, and echocardiographic data obtained at the time of initial diagnosis were retrospectively collected from medical records.

For long-term cardiac evaluation, all patients were prospectively invited for follow-up assessment, and comprehensive transthoracic echocardiography, including speckle-tracking echocardiographic analysis of myocardial deformation parameters, was performed. The control group consisted of age- and sex-matched healthy children without known cardiovascular or systemic disease, who were also prospectively evaluated.

The study was conducted in accordance with the Declaration of Helsinki and was approved by the institutional ethics committee (Approval No. 09.2020.936; Date: September 4, 2020).

### Clinical and laboratory data collection

Acute-phase clinical data, including demographic characteristics, presenting symptoms, vital signs, laboratory parameters, electrocardiographic findings, and echocardiographic measurements obtained at the time of initial diagnosis, were retrospectively collected from hospital records.

Laboratory parameters included cardiac biomarkers (troponin T, creatine kinase [CK], and creatine kinase-MB [CK-MB]), inflammatory markers (C-reactive protein and erythrocyte sedimentation rate), and complete blood count parameters. The neutrophil-to-lymphocyte ratio (NLR) was calculated as the ratio of neutrophil count to lymphocyte count. The systemic immune-inflammation index (SII) was calculated using the following formula:

$$\mathrm{SII}=\frac{\mathrm{platelet}\;\mathrm{count}\times \mathrm{neutrophil}\;\mathrm{count}}{\mathrm{lymphocyte}\;\mathrm{count}}$$

### Echocardiographic assessment

Transthoracic echocardiography was performed using a Philips EPIQ 7 ultrasound system (Philips Healthcare, Andover, MA, USA) by a single experienced operator, in accordance with the recommendations of the American Society of Echocardiography. All images were digitally stored for offline analysis.

M-mode and two-dimensional echocardiographic measurements included interventricular septal thickness in diastole (IVSd), left ventricular end-diastolic diameter (LVDd), left ventricular end-systolic diameter (LVSd), left ventricular posterior wall thickness in diastole (LVPWd), aortic root diameter, and left atrial diameter. Left ventricular systolic function was assessed conventionally using M-mode-derived ejection fraction (EF) and fractional shortening (FS). These measurements were used for the clinical characterization of left ventricular systolic function. Z-scores were calculated according to body surface area using normative data derived from Boston Children's Hospital (Boston Children's Hospital Z-score Calculator, available at: https://zscore.chboston.org/). All measurements were performed three times, and the average values were used for analysis.

Doppler echocardiography was used to evaluate diastolic function. Transmitral flow velocities, including early (E) and late (A) diastolic velocities, deceleration time (DT), and isovolumic relaxation time (IVRT), were measured, and the E/A ratio was calculated.

Tissue Doppler imaging (TDI) was performed at the lateral mitral annulus to obtain myocardial velocities, including systolic (S’), early diastolic (E′), and late diastolic (A′) velocities. Isovolumic contraction time (IVCT), isovolumic relaxation time (IVRT), and ejection time (ET) were measured, and the myocardial performance index (MPI) was calculated using the following formula:

$$\mathrm{MPI}=\frac{\mathrm{IVCT}+\mathrm{IVRT}}{\mathrm{ET}}$$

Two-dimensional speckle-tracking echocardiography was used to assess left ventricular myocardial deformation. Longitudinal strain parameters were obtained from apical two-, three-, and four-chamber views, and global longitudinal strain was calculated. Circumferential strain parameters were obtained from short-axis views at basal, mid-ventricular, and apical levels. Time-to-peak strain dispersion parameters were also recorded. These parameters represented the standard deviation of segmental time-to-peak strain values, were expressed in milliseconds (ms), and were analyzed as absolute time-based measures without normalization to the RR interval or cardiac cycle duration. A bull's-eye plot was generated to evaluate segmental myocardial function.

Two-dimensional images used for speckle-tracking analysis were acquired at frame rates ranging from approximately 50 to 90 frames/s. Tracking quality was visually assessed throughout the cardiac cycle, with particular attention to adequate visualization of the endocardial border, avoidance of apical foreshortening, and appropriate positioning of the myocardial region of interest. When tracking was suboptimal, the region of interest was manually adjusted and the analysis was repeated. Adequate tracking was achieved in all analyzed segments. Positive or markedly extreme strain values were re-evaluated for technical adequacy, including assessment of tracking consistency with myocardial motion and appropriate region-of-interest positioning.

Speckle-tracking analyses were performed offline using the Philips QLAB aCMQ (Automated Cardiac Motion Quantification) software package. During offline aCMQ analysis, the software also automatically generated bi-plane left ventricular end-diastolic volume, end-systolic volume, and ejection fraction. Additional aCMQ-derived parameters included left ventricular end-diastolic and end-systolic areas, fractional area change (FAC), and left ventricular mass. The aCMQ-derived EDV, ESV, and EF were software-generated outputs of the deformation-analysis workflow rather than dedicated clinical measurements of left ventricular systolic function. Because these values depend on the software-specific endocardial tracking, cardiac-cycle timing, and volumetric calculation implemented within the aCMQ workflow, they were interpreted separately from the conventional M-mode-derived systolic function measurements. Accordingly, the aCMQ-derived EF values were not interpreted according to conventional clinical EF thresholds.

### Additional imaging modalities

Cardiac magnetic resonance imaging (CMR) and coronary computed tomography angiography (CCTA) were performed when clinically feasible and indicated as part of the diagnostic evaluation.

CMR was used to assess myocardial involvement and tissue characteristics, including myocardial edema and late gadolinium enhancement. The timing of CMR varied depending on clinical conditions and availability.

CCTA was performed to evaluate coronary artery anatomy, to exclude coronary anomalies or pathologies, and as part of the differential diagnostic work-up when clinically indicated.

### Statistical analysis

Statistical analyses were performed using SPSS software for Windows, version 15.0 (SPSS Inc., Chicago, IL, USA). Descriptive statistics were expressed as number and percentage for categorical variables and as mean ± standard deviation or median (minimum-maximum) for continuous variables.

Comparisons of categorical variables between groups were performed using the chi-square test. Continuous variables were compared using the Student's *t*-test, Mann–Whitney *U*-test, or Friedman test, as appropriate. Correlations between continuous variables were evaluated using Spearman's correlation analysis.

LV global longitudinal strain (GLS) and LV global circumferential strain (GCS) were considered the principal global deformation outcomes, while the corresponding global longitudinal and circumferential aCMQ-derived measures were evaluated as supportive outcomes. Because body weight and body surface area (BSA) differed between groups, separate analysis of covariance (ANCOVA) models were fitted for these four global deformation measures, with group as a fixed factor and BSA as a continuous covariate. Adjusted mean differences (patient minus control) with 95% confidence intervals and partial *η*^2^ values were calculated. Homogeneity of regression slopes was assessed using group-by-BSA interaction terms, and outcome-specific complete-case analyses were performed without imputation.

Given the strong correlation between body weight and BSA, these variables were not entered simultaneously into the same model. Sensitivity analyses were therefore performed using body weight in place of BSA and with additional adjustment for age and sex. Because model residuals showed deviation from normality, robustness of the findings was further assessed using rank-transformed ANCOVA, Huber robust regression, and group-stratified percentile bootstrap analyses with 5,000 resamples.

A Holm adjustment was applied across the four global deformation outcomes. Biomarker-echocardiographic correlation analyses were exploratory and hypothesis-generating, and nominal *p* values are reported for these analyses. All statistical tests were two-sided, and a *p* value < 0.05 was considered statistically significant.

## Results

### Study population and baseline characteristics

A total of 30 pediatric patients with a history of myopericarditis and 22 age- and sex-matched healthy controls were included in the study. There was no statistically significant difference between the groups in terms of age or sex distribution (*p* = 0.463 and *p* = 0.708, respectively). The baseline demographic and clinical characteristics of the study population are summarized in Table 1.

**Table 1:** Study population and baseline characteristics.

Variable	Patients (*n* = 30)	Controls (*n* = 22)	*p*-value
	Mean ± SD; Median (min-max)	Mean ± SD; Median (min-max)	
Age (years)	14.70 ± 2.45; 15.50 (9–17)	13.95 ± 2.79; 14.50 (9–17)	0.463^c^
Height (cm)	167.20 ± 11.29; 170.00 (142–180)	159.41 ± 14.42; 158.50 (134–181)	0.076^c^
Weight (kg)	68.49 ± 19.57; 63.50 (36–110)	52.56 ± 16.72; 54.00 (27.7–90)	0.003^b^
BSA (m^2^)	1.75 ± 0.28; 1.72 (1.20–2.29)	1.52 ± 0.29; 1.52 (1.03–2.09)	0.006^b^
Weight percentile	57.20 ± 31.68; 57 (7–97)	45.75 ± 33.68; 42 (5–99)	0.216^b^
Height percentile	43.70 ± 21.66; 42 (7–86)	53.18 ± 27.87; 54 (6–98)	0.228^c^
Weight z-score	0.48 ± 1.24; 0.21 (−1.28–2.78)	−0.09 ± 1.30; −0.19 (−2–3)	0.115^b^
Height z-score	−0.19 ± 0.71; −0.22 (−1.86–1.18)	0.09 ± 0.97; 0.12 (−1.58–2)	0.224^b^

Sex	n (%)	n (%)	*p*-value
Female	4 (13.3)	4 (18.2)	0.708^a^
Male	26 (86.7)	18 (81.8)	

a Chi-square test.

b Student's *t*-test.

c Mann–Whitney *U*-test.

The mean age of the patient group was 14.70 ± 2.45 years (median: 15.50; range: 9–17), while the control group had a mean age of 13.95 ± 2.79 years (median: 14.50; range: 9–17).

Body weight and body surface area (BSA) were significantly higher in the patient group compared to controls (68.49 ± 19.57 kg vs. 52.56 ± 16.72 kg, *p* = 0.003; and 1.75 ± 0.28 m^2^ vs. 1.52 ± 0.29 m^2^, *p* = 0.006, respectively). There were no statistically significant differences between the groups in terms of height, weight percentile, height percentile, weight z-score, or height *z*-score (all *p* > 0.05).

### Acute-phase clinical, laboratory, and electrocardiographic findings

Chest pain was the most common presenting symptom, occurring in 29 patients (96.7%), and 28 patients (93.3%) required hospitalization. All patients had elevated cardiac troponin T levels, confirming myocardial injury. Etiological investigations identified a specific causative agent in 6 patients, including Chlamydia pneumoniae IgM positivity, parvovirus IgM positivity, rhinovirus/enterovirus detection, and elevated anti-streptococcal antibodies. Electrocardiographic abnormalities were present in 18 patients (60.0%), most commonly ST-segment elevation, and electrocardiographic findings normalized within a mean of 3.39 ± 2.50 days. Detailed acute-phase clinical, laboratory, and electrocardiographic findings are summarized in Table 2.

**Table 2:** Acute clinical, laboratory, and electrocardiographic findings in the patient group.

Variable	Patient Group (*n* = 30)
Clinical Presentation, *n* (%)	
Chest pain	29 (96.7)
Dyspnea	4 (13.8)
Upper respiratory tract infection	17 (56.7)
Gastrointestinal symptoms	6 (20.0)
Other systemic symptoms	5 (17.2)
Hospitalization	
Hospitalized	28 (93.3)
Length of stay (days)	5.60 ± 4.29; 5 (0–20)
Vital Signs at Admission	
Body temperature (°C)	36.82 ± 0.77; 36.65 (35.60–39.50)
Heart rate (beats/min)	84.10 ± 18.98; 83.50 (55–152)
Systolic BP (mmHg)	115.20 ± 10.61; 113 (100–140)
Diastolic BP (mmHg)	71.73 ± 9.65; 71.50 (56–90)
Respiratory rate (breaths/min)	22.00 ± 2.30; 22 (20–28)
Oxygen saturation (%)	99.03 ± 0.93; 99 (96–100)
Laboratory Findings	
Troponin T (baseline)	419.35 ± 409.20; 285.30 (24.94–1,686)
Troponin T (peak)	754.65 ± 634.92; 704.50 (24.94–2,228)
Time to peak troponin (hours)	23.23 ± 14.94; 24 (2–48)
CK (U/L)	348.40 ± 283.97; 225.50 (39–1,137)
CK-MB (baseline)	32.66 ± 39.31; 18.05 (0.30–146)
CK-MB (peak)	57.17 ± 67.10; 38.70 (0.40–298)
Time to peak CK-MB (hours)	16.17 ± 13.34; 14 (1–48)
BNP (pg/mL)	1,002.06 ± 2490.42; 134 (20.40–7,632)
CRP (mg/L)	45.44 ± 56.46; 35.20 (3.10–269)
ESR (mm/h)	24.77 ± 20.35; 20 (1–108)
AST (U/L)	53.89 ± 32.96; 44 (15–143)
ALT (U/L)	23.10 ± 12.64; 20.50 (9–69)
BUN (mg/dL)	11.20 ± 2.93; 12 (5–17)
Creatinine (mg/dL)	0.66 ± 0.14; 0.67 (0.34–0.93)
Hemoglobin (g/dL)	13.40 ± 1.57; 13.25 (8.50–16.80)
WBC (/mm^3^)	9666.67 ± 3271.12; 8,550 (3,900–18,400)
Neutrophil-to-lymphocyte ratio	3.78 ± 2.68; 2.91 (0.85–11.60)
Platelets (/mm^3^)	221766.67 ± 63873.63; 217,500 (115,000–345,000)
Systemic immune-inflammation index (SII)	810389.67 ± 612310.97; 556,740 (137,700–2,510,000)
ECG Findings, *n* (%)	
Normal ECG	12 (40.0)
Any ECG abnormality	18 (60.0)
Inferior ST-segment elevation	12 (40.0)
Anterior/lateral ST-segment elevation	9 (30.0)
T-wave inversion/repolarization abnormalities	3 (10.0)
Diffuse ST-segment elevation	1 (3.3)
Time to ECG normalization (days)	3.39 ± 2.50; 2.50 (1–11)

Categorical variables are presented as number (percentage), and continuous variables as mean ± standard deviation and median (min-max).

BP, blood pressure; BNP, B-type natriuretic peptide; BUN, blood urea nitrogen; CK, creatine kinase; CK-MB, creatine kinase-myocardial band; CRP, C-reactive protein; ECG, electrocardiography; ESR, erythrocyte sedimentation rate; WBC, white blood cell count; AST, aspartate aminotransferase; ALT, alanine aminotransferase. Some patients had more than one electrocardiographic abnormality; therefore, the individual ECG categories are not mutually exclusive.

### Acute-phase echocardiographic findings

At the time of diagnosis, conventional echocardiographic assessment demonstrated largely preserved left ventricular systolic function, with a mean fractional shortening of 38.03 ± 5.92% and a mean ejection fraction of 68.57 ± 6.88%. No valvular regurgitation was detected, and pericardial effusion was present in only 1 patient (3.3%). Detailed acute-phase echocardiographic findings are presented in Table 3.

**Table 3:** Acute echocardiographic findings in the patient group.

Variable	Patients (*n* = 30)
IVSd (cm)	0.89 ± 0.16; 0.93 (0.53–1.10)
LVDd (cm)	4.75 ± 0.43; 4.76 (3.72–5.68)
LVSd (cm)	3.01 ± 0.53; 3.01 (1.91–3.99)
LVPWd (cm)	0.85 ± 0.15; 0.88 (0.49–1.09)
IVSd z-score	0.18 ± 1.23; 0.41 (−2.07–2.90)
LVDd z-score	0.46 ± 1.33; 0.20 (−2.37–3.13)
LVSd z-score	0.07 ± 1.28; 0.35 (−3.00–2.34)
LVPWd z-score	0.29 ± 1.33; 0.45 (−2.36–2.44)
FS (%)	38.03 ± 5.92; 37.50 (26–51)
EF (%)	68.57 ± 6.88; 68.50 (50–81)
LA (cm)	3.00 ± 0.38; 3.03 (2.44–4.10)
LA z-score	0.52 ± 0.92; 0.74 (−1.64–2.40)
Aortic root (cm)	2.47 ± 0.34; 2.46 (1.75–3.09)
Aortic root z-score	−0.22 ± 1.31; −0.28 (−2.29–2.43)
Mitral E (m/s)	0.99 ± 0.22; 0.94 (0.78–1.92)
Mitral A (m/s)	0.56 ± 0.11; 0.56 (0.34–0.83)
Mitral E/A	1.84 ± 0.57; 1.75 (1.06–4.26)
Mitral E z-score	0.44 ± 1.22; 0.28 (−0.79–5.62)
Mitral A z-score	0.96 ± 0.92; 0.97 (−0.76–3.21)
Mitral E/A z-score	−0.63 ± 0.95; −0.85 (−1.96–3.40)
DT (ms)	131.73 ± 36.18; 127.50 (67–201)
DT z-score	−0.47 ± 1.11; −0.75 (−2.44–1.45)
IVRT (ms)	62.93 ± 18.37; 58.50 (25–123)
Aortic velocity (m/s)	1.29 ± 0.18; 1.30 (0.99–1.79)
Pulmonary artery velocity (m/s)	1.18 ± 0.18; 1.15 (0.84–1.63)
Descending aortic velocity (m/s)	1.33 ± 0.15; 1.33 (1.01–1.78)

Continuous variables are presented as mean ± standard deviation and median (min–max).

IVSd, interventricular septal thickness in diastole; LVDd, left ventricular end-diastolic diameter; LVSd, left ventricular end-systolic diameter; LVPWd, left ventricular posterior wall thickness in diastole; FS, fractional shortening; EF, ejection fraction; LA, left atrium; DT, deceleration time; IVRT, isovolumic relaxation time.

### Additional imaging findings

Cardiac magnetic resonance imaging (CMR) was performed in 21 patients. Imaging was obtained within the first 15 days after diagnosis in 14 patients, between 15 and 30 days in 3 patients, and between 3 and 8 months in 4 patients. Pathological findings were detected in 6 patients (28.6%), whereas 15 patients (71.4%) showed no abnormalities. Of the 6 patients with pathological findings, 4 underwent CMR during the acute phase; among these, myocardial edema with late gadolinium enhancement was observed in 2 patients, and pericardial involvement in 2 patients. The remaining 2 patients, who were imaged at 6 and 8 months after diagnosis, showed non-ischemic fibrotic changes.

Coronary computed tomography angiography (CCTA) was performed in 19 patients. Abnormal findings were detected in 2 patients (10.5%), whereas 17 patients (89.5%) showed no coronary pathology. The detected abnormalities included minimal myocardial bridging in the distal left anterior descending artery and hypoplasia of the right coronary artery in a left-dominant system.

### Long-term conventional echocardiographic findings

At a mean follow-up duration of 32.80 ± 11.98 months, fractional shortening and ejection fraction were significantly lower in the patient group than in controls (both *p* < 0.001). Left atrial z-score was significantly higher (*p* = 0.039), whereas deceleration time z-score was significantly lower (*p* = 0.007) in patients. No valvular regurgitation was detected, and pericardial findings were normal in all patients at follow-up. Detailed conventional echocardiographic findings are presented in Table 4.

**Table 4:** Long-term conventional echocardiographic findings in patients and controls.

Variable	Patients (*n* = 30)	Controls (*n* = 22)	*p*-value
IVSd (cm)	0.91 ± 0.14; 0.91 (0.68–1.22)	0.80 ± 0.14; 0.80 (0.54–1.12)	0.005^a^
LVDd (cm)	5.08 ± 0.44; 5.05 (4.22–6.03)	4.55 ± 0.43; 4.50 (3.70–5.40)	<0.001^a^
LVSd (cm)	3.22 ± 0.34; 3.20 (2.63–4.16)	2.69 ± 0.37; 2.65 (1.70–3.50)	<0.001^a^
LVPWd (cm)	0.84 ± 0.13; 0.87 (0.61–1.10)	0.70 ± 0.11; 0.70 (0.54–1.02)	<0.001^a^
IVSd z-score	−0.25 ± 1.02; −0.34 (−1.91–1.99)	−0.56 ± 0.85; −0.55 (−1.81–0.83)	0.254^a^
LVDd z-score	0.31 ± 1.31; −0.19 (−1.43–3.83)	−0.11 ± 0.95; −0.32 (−1.49–2.49)	0.317^b^
LVSd z-score	0.06 ± 1.06; −0.06 (−1.75–3.69)	−0.82 ± 0.91; −1.11 (−1.89–1.03)	0.002^b^
LVPWd z-score	−0.42 ± 1.06; −0.43 (−2.51–1.40)	−1.09 ± 0.73; −1.35 (−1.99–0.51)	0.014^a^
FS (%)	35.10 ± 3.78; 35 (30–43)	39.55 ± 4.06; 39.5 (33–46)	<0.001^a^
EF (%)	64.53 ± 4.91; 64 (56–74)	70.32 ± 4.81; 70 (63–78)	<0.001^a^
LA (cm)	3.36 ± 0.51; 3.36 (2.22–4.40)	2.90 ± 0.37; 2.97 (2.30–3.50)	0.001^a^
LA z-score	0.84 ± 1.28; 0.89 (−1.63–3.00)	0.14 ± 1.02; 0.32 (−2.04–1.45)	0.039^a^
Aortic root (cm)	2.83 ± 0.37; 2.89 (1.87–3.43)	2.47 ± 0.33; 2.39 (1.98–3.30)	0.001^a^
Aortic root z-score	0.18 ± 1.25; 0.34 (−2.35–3.18)	−0.31 ± 1.03; −0.31 (−2.23–1.85)	0.139^a^
Mitral E (m/s)	1.03 ± 0.15; 1.00 (0.76–1.41)	0.99 ± 0.16; 0.98 (0.68–1.33)	0.272^a^
Mitral A (m/s)	0.66 ± 0.18; 0.67 (0.38–1.31)	0.61 ± 0.14; 0.61 (0.33–0.84)	0.384^b^
DT (ms)	137.77 ± 46.14; 135.5 (60–232)	166.91 ± 33.50; 166 (114–232)	0.015^a^
Mitral E z-score	0.66 ± 0.92; 0.42 (−0.98–2.81)	0.24 ± 0.94; 0.21 (−1.37–2.33)	0.120^a^
Mitral A z-score	1.87 ± 1.45; 1.90 (−0.41–7.11)	1.44 ± 1.14; 1.42 (−0.80–3.24)	0.354^b^
DT z-score	−0.58 ± 1.24; −0.50 (−2.74–2.10)	0.31 ± 0.98; 0.10 (−1.04–2.81)	0.007^a^
IVRT (ms)	59.87 ± 11.10; 61.5 (35–81)	62.09 ± 11.69; 65 (37–81)	0.488^a^
Mitral E/A	1.64 ± 0.39; 1.55 (0.92–2.52)	1.65 ± 0.38; 1.58 (1.15–3.03)	0.956^b^
Mitral E/A z-score	−1.02 ± 0.63; −1.20 (−2.24–0.38)	−1.00 ± 0.59; −1.09 (−1.67–1.16)	0.897^a^
Aortic velocity (m/s)	1.44 ± 0.24; 1.49 (1.02–1.87)	1.30 ± 0.23; 1.29 (0.96–1.80)	0.047^a^
Pulmonary artery velocity (m/s)	1.25 ± 0.18; 1.18 (0.97–1.63)	1.13 ± 0.18; 1.13 (0.81–1.50)	0.018^a^
Descending aortic velocity (m/s)	1.52 ± 0.23; 1.50 (1.11–2.23)	1.36 ± 0.23; 1.36 (1.04–1.80)	0.018^a^

Continuous variables are presented as mean ± standard deviation and median (min-max).

a Student's *t*-test.

b Mann–Whitney *U*-test.

IVSd, interventricular septal thickness in diastole; LVDd, left ventricular end-diastolic diameter; LVSd, left ventricular end-systolic diameter; LVPWd, left ventricular posterior wall thickness in diastole; FS, fractional shortening; EF, ejection fraction; LA, left atrium; DT, deceleration time; IVRT, isovolumic relaxation time.

### Tissue Doppler findings

At follow-up, E′ and A′ velocities were significantly higher in the patient group compared with controls (both *p* = 0.008). E′ and A′ z-scores were also significantly higher in patients (*p* = 0.011 and *p* = 0.008, respectively). Detailed tissue Doppler parameters are presented in Table 5.

**Table 5:** Advanced echocardiographic parameters in patients and controls.

Variable	Patients (*n* = 30)	Controls (*n* = 22)	*p*-value
Tissue Doppler parameters			
S’	12.00 ± 2.44; 11.75 (7.29–17.30)	10.56 ± 2.94; 10.25 (6.60–20.00)	0.060^a^
E′	18.72 ± 3.81; 17.60 (12.5–27.2)	16.35 ± 2.34; 16.75 (11.9–21.0)	0.008^a^
A′	8.45 ± 2.32; 8.54 (4.42–13.00)	6.92 ± 1.69; 7.13 (3.87–11.40)	0.008^a^
E′/A′	2.32 ± 0.58; 2.30 (1.27–3.63)	2.42 ± 0.68; 2.26 (0.83–3.50)	0.572^a^
S′ z-score	0.54 ± 1.00; 0.45 (−1.54–2.64)	−0.01 ± 1.21; −0.23 (−1.79–3.77)	0.081^a^
E′ z-score	−0.07 ± 1.27; −0.20 (−2.16–2.73)	−0.82 ± 0.75; −0.65 (−2.32–0.56)	0.011^a^
A′ z-score	1.62 ± 1.39; 1.69 (−0.79–4.33)	0.71 ± 1.02; 0.84 (−1.10–3.38)	0.008^a^
E′/A′ z-score	−1.06 ± 0.49; −1.10 (−1.92–0.00)	−1.00 ± 0.58; −1.12 (−2.53–0.00)	0.689^a^
MPI	0.46 ± 0.09; 0.46 (0.32–0.81)	0.51 ± 0.21; 0.48 (0.31–1.28)	0.559^b^
E/E′	0.06 ± 0.01; 0.06 (0.04–0.09)	0.06 ± 0.02; 0.06 (0.04–0.14)	0.143^b^
MAPSE	18.03 ± 1.99; 18.00 (11.0–20.9)	17.91 ± 2.74; 18.85 (11.8–22.0)	0.792^b^
Longitudinal strain and time-to-peak parameters			
LV EDV (aCMQ bi-plane, mL)	110.25 ± 24.73; 109.10 (66.4–160.1)	84.65 ± 26.21; 77.45 (48.6–133.2)	0.001^a^
LV ESV (aCMQ bi-plane, mL)	67.35 ± 19.81; 66.35 (36.9–111.9)	47.46 ± 17.33; 43.45 (25.4–84.4)	<0.001^b^
LV EF (aCMQ bi-plane, %)	39.63 ± 6.62; 39.05 (26.8–50.1)	44.61 ± 5.27; 46.00 (32.4–52.8)	0.006^a^
LV AP2 endo peak L time SD (ms)	103.92 ± 139.30; 51.05 (25.9–591.5)	63.29 ± 30.71; 53.45 (27.8–129.1)	0.919^b^
LV AP3 endo peak L time SD (ms)	101.51 ± 120.12; 61.70 (25.4–468.4)	50.58 ± 29.14; 38.90 (17.9–137.0)	0.008^a^
LV AP4 endo peak L time SD (ms)	100.78 ± 126.33; 38.80 (15.8–493.0)	45.86 ± 20.95; 40.75 (15.9–101.3)	0.236^a^
LV global endo peak L time SD (ms)	71.49 ± 168.15; 16.75 (1.6–703.3)	17.91 ± 12.16; 14.80 (3.3–40.9)	0.511^a^
LV AP2 endo peak L strain	−13.02 ± 8.16; −15.00 (−21.6–18.1)	−17.13 ± 2.41; −17.65 (−21.0–−11.8)	0.009^a^
LV AP3 endo peak L strain	−12.65 ± 7.80; −13.35 (−23.1–16.0)	−17.77 ± 2.88; −17.70 (−22.9–−12.5)	0.005^a^
LV AP4 endo peak L strain	−11.70 ± 9.30; −14.15 (−20.1–18.0)	−18.40 ± 2.55; −18.25 (−22.8–−13.1)	0.001^a^
LV global endo peak L strain	−12.43 ± 6.67; −14.35 (−19.6–7.0)	−17.77 ± 1.94; −17.60 (−21.7–−14.1)	<0.001^a^
Circumferential strain and time-to-peak parameters			
LV SAX A endo peak C time SD (ms)	73.26 ± 109.63; 35.60 (1.4–456.4)	21.10 ± 16.90; 14.85 (1.7–60.8)	0.008^a^
LV SAX M endo peak C time SD (ms)	66.31 ± 89.97; 36.15 (5.6–449.8)	35.80 ± 26.37; 28.15 (7.8–108.7)	0.003^a^
LV SAX B endo peak C time SD (ms)	139.47 ± 176.39; 69.50 (9.1–658.0)	46.06 ± 25.31; 41.05 (5.0–109.1)	0.017^a^
LV global endo peak C time SD (ms)	98.68 ± 193.78; 28.00 (2.8–686.0)	24.54 ± 18.54; 22.15 (2.5–61.8)	0.154^b^
LV SAX A endo peak C strain	−19.03 ± 15.46; −23.30 (−39.1–32.1)	−30.32 ± 8.81; −28.80 (−50.3–−17.8)	0.003^a^
LV SAX M endo peak C strain	−13.66 ± 16.45; −19.30 (−31.4–33.1)	−24.09 ± 4.90; −25.45 (−30.6–−13.8)	0.109^b^
LV SAX B endo peak C strain	−14.00 ± 13.09; −16.90 (−29.5–21.7)	−22.74 ± 5.71; −21.65 (−36.1–−13.2)	0.005^a^
LV global endo peak C strain	−16.04 ± 12.50; −19.80 (−27.3–27.7)	−25.14 ± 4.73; −25.65 (−31.4–−10.8)	0.002^a^
aCMQ software-derived myocardial deformation parameters			
LV global endo L peak ACMQ	−12.46 ± 6.71; −14.32 (−19.57–7.04)	−17.70 ± 1.96; −17.48 (−21.71–−14.12)	0.001^a^
LV AP4 endo L peak ACMQ	−10.81 ± 10.18; −14.23 (−20.99–18.01)	−18.41 ± 2.54; −18.24 (−22.78–−13.18)	<0.001^b^
LV AP2 endo L peak ACMQ	−12.17 ± 9.81; −14.91 (−21.91–18.09)	−17.30 ± 2.30; −17.99 (−21.00–−11.84)	0.007^b^
LV AP3 endo L peak ACMQ	−11.91 ± 9.00; −13.36 (−23.11–16.04)	−17.78 ± 2.88; −17.67 (−22.95–−12.52)	0.001^b^
LV global endo C peak ACMQ	−18.34 ± 7.39; −19.97 (−26.33–6.91)	−25.73 ± 3.56; −25.65 (−31.45–−19.28)	<0.001^a^
LV SAX B endo C peak ACMQ	−14.90 ± 11.79; −16.78 (−29.55–17.54)	−22.74 ± 5.72; −21.69 (−36.12–−13.18)	0.006^a^
LV SAX M endo C peak ACMQ	−15.89 ± 13.07; −20.02 (−29.19–24.34)	−24.11 ± 4.89; −25.44 (−30.63–−13.81)	0.008^a^
LV SAX A endo C peak ACMQ	−19.72 ± 16.36; −23.74 (−45.66–32.10)	−30.34 ± 8.80; −28.85 (−50.27–−17.77)	0.008^a^
Area and mass parameters			
LV EDA (cm^2^)	18.80 ± 4.16; 18.72 (12.04–27.55)	14.21 ± 3.16; 13.93 (8.29–20.66)	<0.001^a^
LV ESA (cm^2^)	11.81 ± 2.96; 11.60 (7.39–18.65)	7.97 ± 2.19; 7.33 (3.75–11.90)	<0.001^a^
LV FAC (%)	37.14 ± 7.89; 38.56 (17.96–50.05)	44.27 ± 7.63; 46.40 (26.88–54.77)	0.003^a^
LV mass (cubed), g	161.07 ± 37.23; 155 (99–240)	109.45 ± 33.53; 102 (70–212)	<0.001^a^

Continuous variables are presented as mean ± standard deviation and median (min-max).

a Student's *t*-test.

b Mann–Whitney *U*-test.

MPI, myocardial performance index; MAPSE, mitral annular plane systolic excursion; EDV, end-diastolic volume; ESV, end-systolic volume; EF, ejection fraction; LV, left ventricle; AP2, apical 2-chamber; AP3, apical 3-chamber; AP4, apical 4-chamber; SAX, short-axis; L, longitudinal; C, circumferential; aCMQ, Automated Cardiac Motion Quantification software package used for speckle-tracking analysis; EDA, end-diastolic area; ESA, end-systolic area; FAC, fractional area change. Time SD: standard deviation of segmental time-to-peak strain values; values are expressed in milliseconds (ms). The aCMQ-derived bi-plane volumetric parameters are software-generated outputs of the offline aCMQ analysis and were not used for the conventional assessment of left ventricular systolic function reported in Table 4. These software-derived EF values should therefore not be interpreted according to conventional clinical EF thresholds.

### Longitudinal strain analysis

At follow-up, global longitudinal strain was significantly reduced in the patient group compared with controls (−12.43 ± 6.67% vs. −17.77 ± 1.94%, *p* < 0.001). Segmental analysis showed that longitudinal strain values in the apical 2-chamber, apical 3-chamber, and apical 4-chamber views were also significantly lower in the patient group (*p* = 0.009, *p* = 0.005, and *p* = 0.001, respectively). A significant difference was observed in apical 3-chamber longitudinal time-to-peak dispersion (Time SD) (*p* = 0.008), whereas the corresponding apical 2-chamber, apical 4-chamber, and global longitudinal time-to-peak dispersion parameters did not differ significantly between the groups (all *p* > 0.05). Detailed longitudinal strain parameters are presented in Table 5.

### Circumferential strain and area-based functional parameters

Global circumferential strain was significantly lower in the patient group compared with controls (−16.04 ± 12.50% vs. −25.14 ± 4.73%, *p* = 0.002). Segmental analysis showed significantly lower circumferential strain values at the apical and basal levels (*p* = 0.003 and *p* = 0.005, respectively), whereas the mid-ventricular difference was not statistically significant (*p* = 0.109).

Circumferential time-to-peak dispersion parameters were significantly higher in the patient group at the apical, mid, and basal levels (*p* = 0.008, *p* = 0.003, and *p* = 0.017, respectively), while global circumferential time-to-peak dispersion did not differ significantly between groups (*p* = 0.154).

Additional aCMQ-derived myocardial deformation measures showed a similar pattern, with significantly lower global longitudinal and circumferential deformation values in patients compared with controls (−12.46 ± 6.71% vs. −17.70 ± 1.96%, *p* = 0.001; and −18.34 ± 7.39% vs. −25.73 ± 3.56%, *p* < 0.001, respectively). Segmental aCMQ-derived longitudinal and circumferential deformation values were also significantly lower in the patient group.

Left ventricular end-diastolic and end-systolic areas were significantly higher, whereas fractional area change was significantly lower in patients; left ventricular mass was also higher. Detailed circumferential strain, aCMQ-derived deformation, and area-based parameters are presented in Table 5.

### BSA-adjusted global myocardial deformation analysis

After adjustment for BSA, the between-group differences remained statistically significant for the principal global deformation outcomes and both supportive aCMQ-derived measures. The adjusted mean difference was +4.17 for GLS (95% CI, 1.08–7.26; *p* = 0.009), +7.39 for GCS (95% CI, 1.38–13.41; *p* = 0.017), +4.09 for global longitudinal peak aCMQ (95% CI, 0.92–7.27; *p* = 0.013), and +6.93 for global circumferential peak aCMQ (95% CI, 3.21–10.64; *p* < 0.001). Group-by-BSA interactions were not significant for any of the four global measures (*p* = 0.227–0.659). The findings were consistent after replacing BSA with body weight, after additional adjustment for age and sex, and in rank-transformed ANCOVA, Huber robust regression, and bootstrap sensitivity analyses. All four global outcomes also remained statistically significant after Holm adjustment for multiple comparisons (maximum adjusted *p* = 0.027).

Detailed results of the BSA-adjusted global deformation analyses and corresponding sensitivity analyses are provided in Supplementary Table S1.

### Regional bull's-eye strain analysis

Segment-wise analysis of available bull's-eye strain data did not demonstrate a statistically significant regional predominance of strain abnormalities. For longitudinal strain, mean impairment tended to be greater at the basal level compared with the mid and apical levels (−13.94 ± 7.21%, −11.53 ± 5.85%, and −10.59 ± 5.36%, respectively; *p* = 0.057). For circumferential strain, mean impairment was numerically greater at the apical level (−18.80 ± 10.09%) than at the mid (−17.24 ± 10.31%) and basal (−15.45 ± 8.30%) levels, although this difference was not statistically significant (*p* = 0.214) (Supplementary Table S2). Representative tissue Doppler imaging and longitudinal and circumferential bull's-eye strain maps are shown in Figure 1.

**Figure 1:**
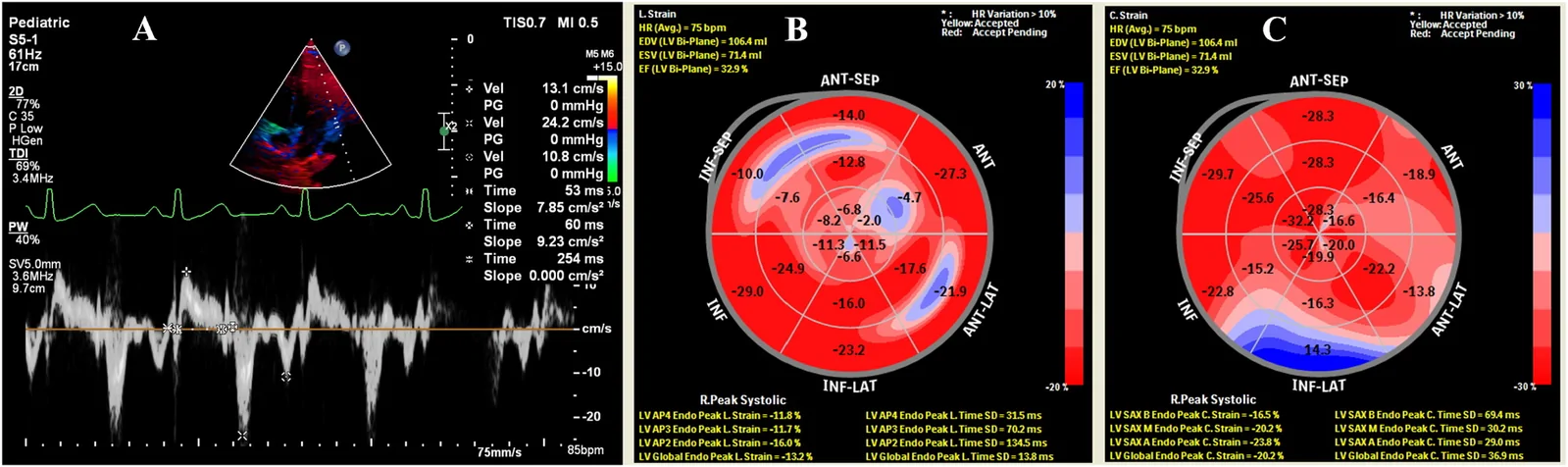
Representative echocardiographic findings in a pediatric patient with previous myopericarditis. **(A)** Tissue Doppler imaging obtained from the lateral mitral annulus. **(B)** Left ventricular longitudinal strain bull's-eye map demonstrating heterogeneous regional myocardial deformation abnormalities. **(C)** Left ventricular circumferential strain bull's-eye map demonstrating circumferential myocardial deformation abnormalities.

### Correlation analyses between acute-phase markers and long-term echocardiographic parameters

#### Correlations with conventional echocardiographic parameters

Exploratory analyses identified several associations between acute-phase biomarkers and long-term conventional echocardiographic parameters. Peak troponin T, CK-MB, BNP, and CRP showed significant correlations with selected structural and diastolic measures. In particular, CRP was negatively correlated with deceleration time (*r* = −0.457, *p* = 0.011) and DT z-score (*r* = −0.492, *p* = 0.006).

#### Correlations with tissue Doppler, strain, and myocardial deformation parameters

NLR and SII were associated primarily with tissue Doppler and longitudinal deformation measures. NLR was positively correlated with global longitudinal strain (*r* = 0.437, *p* = 0.016), while SII was positively correlated with global longitudinal strain (*r* = 0.456, *p* = 0.011) and global longitudinal deformation (*r* = 0.452, *p* = 0.012). CRP was positively correlated with global circumferential strain (*r* = 0.406, *p* = 0.029). Detailed results of all exploratory correlation analyses are presented in Supplementary Table S3.

## Discussion

In this study, we performed a comprehensive long-term echocardiographic evaluation in pediatric patients with a history of myopericarditis using conventional echocardiography, tissue Doppler imaging, and two-dimensional speckle-tracking echocardiography. Although conventional systolic function parameters were largely preserved, patients demonstrated significant abnormalities in longitudinal and circumferential myocardial deformation parameters compared with healthy controls. In addition, increased time-to-peak strain dispersion suggested greater heterogeneity in myocardial contraction timing. Exploratory analyses also identified associations between acute-phase inflammatory and cardiac biomarkers, including troponin, CRP, NLR, and SII, and selected long-term echocardiographic and myocardial deformation parameters. Together, these findings suggest that subtle myocardial deformation abnormalities may be detectable at long-term follow-up in pediatric patients with a history of myopericarditis despite generally preserved conventional echocardiographic findings. Beyond the long-term abnormalities observed in longitudinal strain, our findings extend previous observations by demonstrating concomitant circumferential deformation abnormalities and increased time-to-peak strain dispersion in pediatric myopericarditis. The observed associations between acute-phase inflammatory biomarkers and long-term deformation parameters suggest a potential relationship between inflammatory burden and later myocardial mechanical findings; however, the present study cannot determine whether this relationship is causal.

Conventional echocardiographic parameters such as ejection fraction and fractional shortening are widely used for the assessment of ventricular systolic function; however, these parameters may remain within normal limits despite subtle myocardial injury or early impairment in myocardial mechanics. Two-dimensional speckle-tracking echocardiography has emerged as a more sensitive technique for detecting subclinical myocardial dysfunction through quantitative assessment of myocardial deformation (5, 10). In the present study, although conventional systolic function parameters were largely preserved at long-term follow-up, patients with a history of myopericarditis demonstrated significant abnormalities in both longitudinal and circumferential strain parameters compared with healthy controls. These findings are consistent with previous studies demonstrating that strain imaging may reveal myocardial deformation abnormalities despite preserved ejection fraction (5, 6, 11, 12). Similarly, Chinali et al. reported that speckle-tracking echocardiography could detect regional myocardial abnormalities in pediatric focal myocarditis despite normal conventional systolic function (12). Our findings further support the growing evidence that advanced myocardial deformation analysis may provide incremental information beyond conventional echocardiographic parameters during long-term follow-up of pediatric inflammatory myocardial diseases.

The marked difference between conventional M-mode-derived EF and the automatically generated aCMQ bi-plane EF warrants specific consideration. Notably, similarly low aCMQ-derived EF values were also observed in the healthy control group, arguing against interpretation of these values as evidence of disease-specific severe systolic dysfunction. This discrepancy likely reflects methodological characteristics of the aCMQ workflow, including software-specific endocardial tracking, cardiac-cycle timing, and volumetric calculation. Intervendor and software-dependent variability in speckle-tracking measurements is well recognized and has led to international efforts to standardize deformation imaging (13). Accordingly, in the present study, the aCMQ-derived EDV, ESV, and EF were regarded as secondary software-generated analytical outputs rather than dedicated clinical measures of left ventricular systolic function. The aCMQ-derived EF values should therefore not be interpreted according to conventional clinical EF thresholds, and the description of largely preserved systolic function refers specifically to the conventional echocardiographic assessment.

In addition to impaired longitudinal strain parameters, patients in our study also demonstrated significant abnormalities in circumferential strain measurements and time-to-peak strain dispersion. These findings may indicate that myocardial involvement in pediatric myopericarditis is not limited to isolated longitudinal fiber dysfunction but may also affect more complex myocardial mechanics. Since circumferential myocardial fibers are predominantly located within the mid-wall myocardial layers, impairment in circumferential deformation may reflect more diffuse myocardial involvement or alterations in myocardial structure and mechanics during long-term follow-up. Furthermore, increased time-to-peak strain dispersion may suggest greater heterogeneity in myocardial contraction timing. Whether this finding is related to underlying myocardial tissue alterations, including fibrosis or other post-inflammatory changes, remains uncertain and cannot be established from the present study because CMR was not systematically performed and its timing was heterogeneous. Previous studies have demonstrated longitudinal and, to a lesser extent, circumferential deformation abnormalities in myocarditis despite preserved conventional systolic function (11, 12, 14, 15). However, data regarding circumferential deformation and time-to-peak strain parameters in pediatric myopericarditis remain limited. Therefore, our findings may extend the existing evidence regarding long-term myocardial mechanics in pediatric inflammatory myocardial diseases. Furthermore, regional bull's-eye strain analysis did not demonstrate a statistically significant predominance of myocardial deformation abnormalities at any specific myocardial level. Although longitudinal strain impairment tended to be greater at the basal level and circumferential strain impairment appeared numerically more pronounced at the apical level, these differences did not reach statistical significance. These findings indicate that myocardial deformation abnormalities observed at long-term follow-up after pediatric myopericarditis do not appear to follow a consistent regional pattern and may instead be distributed heterogeneously. This observation may reflect the variable extent and localization of myocardial involvement during the acute inflammatory process.

Because body weight and BSA were higher in the patient group, body size was considered a potential confounding factor in the assessment of myocardial deformation. However, the between-group differences in the principal global deformation measures remained statistically significant after adjustment for BSA and were consistent across sensitivity analyses. These findings suggest that the observed differences in global myocardial deformation were not attributable solely to the between-group imbalance in body size.

Tissue Doppler imaging findings in our study also suggested subtle alterations in myocardial function during long-term follow-up. Although conventional diastolic parameters were largely preserved, patients demonstrated significantly higher E′ and A′ velocities as well as increased E′ and A′ z-scores compared with healthy controls. These findings may reflect adaptive or compensatory myocardial functional changes following the acute inflammatory process rather than overt diastolic dysfunction. In contrast, E′/A′ ratio and myocardial performance index did not differ significantly between the groups, suggesting relative preservation of global diastolic function. Previous studies in pediatric inflammatory and systemic diseases have similarly demonstrated that tissue Doppler and myocardial deformation imaging may reveal subtle myocardial functional alterations even in the absence of overt conventional echocardiographic abnormalities (8, 16, 17).

In exploratory analyses, acute-phase cardiac and inflammatory biomarkers were associated with several long-term myocardial deformation and ventricular filling parameters. Troponin T levels were inversely correlated with mitral E velocity and descending aortic flow velocity, while CRP showed correlations with deceleration time indices, circumferential strain parameters, and left ventricular end-diastolic volume. These findings may indicate a potential relationship between the severity of acute myocardial injury or inflammatory activity and long-term myocardial deformation findings after pediatric myopericarditis. Previous studies have similarly demonstrated associations between inflammatory myocardial injury, impaired myocardial deformation, and long-term GLS abnormalities in inflammatory pediatric cardiac diseases (15, 18). Therefore, our findings add to the existing literature by identifying exploratory associations between acute inflammatory burden and myocardial deformation findings observed during long-term follow-up.

In contrast to conventional echocardiographic parameters, NLR and SII in our study were primarily associated with advanced echocardiographic indices, particularly tissue Doppler and myocardial deformation parameters. These findings suggest that associations with systemic inflammatory activation during the acute phase were more apparent in advanced echocardiographic indices than in conventional measurements. Notably, both NLR and SII were associated with impaired longitudinal deformation parameters, while SII also demonstrated relationships with additional speckle-tracking-derived myocardial deformation indices. Recent studies have increasingly highlighted the potential clinical relevance of systemic inflammatory indices, including NLR and SII, in pediatric myocarditis and other inflammatory myocardial diseases (11, 19). However, data regarding the relationship between these inflammatory markers and long-term myocardial deformation findings in pediatric myopericarditis remain limited. Therefore, our findings may provide additional insight into the potential relationship between acute systemic inflammatory burden and subclinical myocardial deformation abnormalities observed during long-term follow-up after pediatric myopericarditis.

Pericardial effusion was uncommon in our cohort and was detected in only one patient during the acute phase. However, the presence of pericardial effusion was not a prerequisite for the clinical diagnosis of myopericarditis, as acute pericarditis may occur with or without pericardial effusion (20). The low prevalence of effusion in our cohort may therefore reflect a clinical phenotype in which pericardial inflammation was predominantly manifested by characteristic chest pain rather than by detectable fluid accumulation.

Cardiac magnetic resonance imaging remains an important modality for confirming myocardial involvement and characterizing myocardial edema, fibrosis, and late gadolinium enhancement in patients with suspected myocarditis or myopericarditis (21). In our cohort, CMR was performed when imaging was feasible, and abnormal findings were detected in a limited proportion of those who underwent CMR. However, the timing of CMR was heterogeneous, ranging from the acute phase to several months after diagnosis. Variability in imaging timing was mainly related to real-world clinical and logistical factors, including patient intolerance, efforts to avoid additional sedation, technical limitations, and referral-related scheduling delays. The negative CMR findings in the remaining patients may therefore reflect the heterogeneous timing of imaging and the limited detectability of transient or less extensive myocardial inflammatory changes, particularly when imaging was performed outside the acute phase. Therefore, no formal correlation analysis between CMR findings and long-term deformation parameters was performed. Nevertheless, myocardial deformation abnormalities detected during long-term echocardiographic follow-up suggest that speckle-tracking echocardiography may provide complementary functional information beyond conventional systolic assessment, particularly in patients with preserved conventional systolic function. Previous studies comparing STE and CMR findings in myocarditis have similarly suggested that strain imaging may detect subtle myocardial abnormalities even in the absence of overt conventional systolic dysfunction (12).

Coronary computed tomography angiography was performed in selected patients in our cohort, particularly to exclude coronary anomalies or other structural coronary pathologies that may mimic myopericarditis in children presenting with chest pain, electrocardiographic abnormalities, and elevated cardiac biomarkers. Most patients demonstrated normal coronary anatomy, while only a limited number of cases showed minor incidental findings. Previous expert consensus documents regarding pediatric coronary artery anomalies have emphasized the role of advanced coronary imaging modalities when coronary anatomy cannot be adequately evaluated by echocardiography or when alternative coronary pathologies are suspected (22).

Patients with pediatric myopericarditis are generally considered to have favorable short-term outcomes, particularly when conventional systolic function normalizes during follow-up. However, our findings suggest that subtle myocardial functional abnormalities may be detectable during long-term follow-up despite apparently preserved conventional echocardiographic parameters. In this context, advanced echocardiographic techniques such as speckle-tracking echocardiography may provide incremental information regarding long-term myocardial deformation beyond conventional systolic measurements alone. The detection of subclinical myocardial deformation abnormalities during long-term follow-up may contribute to a more comprehensive assessment of myocardial mechanics in pediatric myopericarditis. To our knowledge, this study provides one of the more comprehensive long-term echocardiographic evaluations of pediatric myopericarditis, incorporating conventional echocardiography, tissue Doppler imaging, detailed myocardial deformation analysis, and correlations with acute-phase inflammatory and cardiac biomarkers. Although several deformation parameters differed significantly between patients and controls, the clinical significance of these statistical differences remains uncertain because clinical outcomes, exercise capacity, and arrhythmia-related endpoints were not assessed. Their long-term clinical and prognostic implications therefore require clarification in larger prospective studies.

This study has several limitations that should be considered while interpreting the findings. First, this was a single-center study with a relatively small sample size, which may limit the generalizability of the results. Given the multiple exploratory correlation analyses, the possibility of type I error cannot be completely excluded; therefore, the biomarker-echocardiographic associations should be interpreted cautiously and considered hypothesis-generating. Second, although long-term echocardiographic evaluations were prospectively performed, acute-phase clinical and laboratory data were retrospectively collected from medical records. Third, speckle-tracking echocardiography was not available during the acute phase; therefore, temporal changes in myocardial deformation parameters from the acute presentation to long-term follow-up could not be evaluated. In addition, cardiac magnetic resonance imaging was not performed in all patients and the timing of CMR acquisition was heterogeneous, largely because of clinical conditions, technical limitations, and difficulties related to patient cooperation in pediatric imaging. Therefore, direct comparisons between CMR findings and long-term myocardial deformation parameters were not performed. Furthermore, strain measurements were obtained using a single vendor-specific software platform, which may limit the reproducibility of myocardial deformation parameters across different imaging systems. Intraobserver and interobserver reproducibility analyses of the speckle-tracking measurements were not available and should therefore be considered a limitation of the present study.

## Conclusion

Pediatric patients with a history of myopericarditis may demonstrate subclinical myocardial deformation abnormalities during long-term follow-up despite largely preserved conventional echocardiographic findings. Advanced echocardiographic techniques, particularly speckle-tracking echocardiography, may provide additional information regarding myocardial deformation beyond conventional systolic parameters alone. Furthermore, exploratory associations were observed between acute-phase inflammatory and cardiac biomarkers and long-term myocardial deformation parameters. Larger prospective studies incorporating serial multimodality imaging are needed to confirm these associations and to clarify their clinical significance and temporal evolution after pediatric myopericarditis.

## Data Availability

The raw data supporting the conclusions of this article will be made available by the authors, without undue reservation.
