# Supplementary material for: Long-term myocardial deformation abnormalities and biomarker associations in pediatric myopericarditis: a speckle-tracking echocardiographic study
Source: Front Cardiovasc Med. 2026 Oct 28;13:1938858. doi: 10.3389/fcvm.2026.1938858 (PMC13751981; doi:10.3389/fcvm.2026.1938858)
Supplement: Supplementary file 1 [file Table1.docx]

| **Supplementary Table S1. BSA-adjusted analyses of global myocardial deformation measures** | | | | | | |
| --- | --- | --- | --- | --- | --- | --- |
| **Panel A. Unadjusted and BSA-adjusted between-group differences** | | | | | | |
| **Parameter** | **n (Patient/Control)** | **Unadjusted difference (95% CI)** | **BSA-adjusted difference (95% CI)** | **ANCOVA p-value** | **Partial η²** | **Adjusted mean (Patient / Control)** |
| **LV global endo peak L strain (GLS)** | 30 / 22 | +5.35 (2.74, 7.96) | +4.17 (1.08, 7.26) | 0.009 | 0.131 | −12.93 / −17.11 |
| **LV global endo peak C strain (GCS)** | 29 / 22 | +9.10 (3.98, 14.22) | +7.39 (1.38, 13.41) | 0.017 | 0.113 | −16.78 / −24.17 |
| **LV global endo L peak aCMQ** | 30 / 21 | +5.24 (2.61, 7.87) | +4.09 (0.92, 7.27) | 0.013 | 0.123 | −12.96 / −17.05 |
| **LV global endo C peak aCMQ** | 26 / 22 | +7.39 (4.08, 10.71) | +6.93 (3.21, 10.64) | <0.001 | 0.238 | −18.50 / −25.43 |
| **Panel B. Model assumptions and sensitivity analyses** | | | | | | |
| **Parameter** | **BSA effect p-value** | **Group×BSA p-value** | **Bootstrap 95% CI (5,000 resamples)** | **Rank-ANCOVA p-value** | **Huber p-value** | **Additional adjustment for age and sex: difference (95% CI); p-value** |
| **GLS** | 0.048 | 0.521 | 2.10–6.60 | <0.001 | <0.001 | +4.34 (1.12–7.56); p=0.009 |
| **GCS** | 0.135 | 0.227 | 3.05–11.96 | <0.001 | 0.001 | +7.06 (0.75–13.38); p=0.029 |
| **L peak aCMQ** | 0.054 | 0.538 | 1.95–6.45 | <0.001 | <0.001 | +4.27 (0.97–7.56); p=0.012 |
| **C peak aCMQ** | 0.463 | 0.659 | 4.20–9.86 | <0.001 | <0.001 | +6.84 (2.95–10.74); p=0.001 |

Difference = patient − control. Adjusted means were estimated at a common reference BSA of 1.65 m². Unadjusted differences are provided for descriptive comparison. All four global outcomes remained statistically significant after Holm adjustment for multiple comparisons; the largest Holm-adjusted p-value was 0.027. Abbreviations: BSA, body surface area; GLS, global longitudinal strain; GCS, global circumferential strain; aCMQ, automated Cardiac Motion Quantification; ANCOVA, analysis of covariance; CI, confidence interval; LV, left ventricle.
