# Supplementary material for: Long-term myocardial deformation abnormalities and biomarker associations in pediatric myopericarditis: a speckle-tracking echocardiographic study
Source: Front Cardiovasc Med. 2026 Oct 28;13:1938858. doi: 10.3389/fcvm.2026.1938858 (PMC13751981; doi:10.3389/fcvm.2026.1938858)
Supplement: Supplementary file 2 [file Table2.docx]

| **Supplementary Table S2. Regional distribution of longitudinal and circumferential strain values based on bull's-eye analysis** | | |  |
| --- | --- | --- | --- |
| **Myocardial level** | **Longitudinal strain (%), Mean ± SD** | **Circumferential strain(%),**  **Mean ± SD** |  |
|  |  |  |  |
| **Basal** | −13.94 ± 7.21 | −15.45 ± 8.30 |  |
| **Mid-ventricular** | −11.53 ± 5.85 | −17.24 ± 10.31 |  |
| **Apical** | −10.59 ± 5.36 | −18.80 ± 10.09 |  |
| **p value†** | 0.057 | 0.214 |  |

† Friedman test comparing basal, mid-ventricular, and apical myocardial levels.
