# Supplementary material for: Long-term myocardial deformation abnormalities and biomarker associations in pediatric myopericarditis: a speckle-tracking echocardiographic study
Source: Front Cardiovasc Med. 2026 Oct 28;13:1938858. doi: 10.3389/fcvm.2026.1938858 (PMC13751981; doi:10.3389/fcvm.2026.1938858)
Supplement: Supplementary file 3 [file Table3.docx]

| **Supplementary Table S3. Correlation of Acute-Phase Biomarkers with Conventional and Advanced Echocardiographic Parameters** | | | | | | | | | | | | |
| --- | --- | --- | --- | --- | --- | --- | --- | --- | --- | --- | --- | --- |
| **Variable** | **Peak Trp T** | | **Peak CK-MB** | | **BNP** | | **CRP** | | **NLR** | | **SII** | |
|  | r | p | r | p | r | p | r | p | r | p | r | p |
| **Conventional  Echocardiographic Parameters** |  |  |  |  |  |  |  |  |  |  |  |  |
| IVSd (cm) | 0.252 | 0.179 | 0.246 | 0.190 | -0.511 | 0.160 | 0.081 | 0.669 | 0.012 | 0.950 | -0.082 | 0.667 |
| LVDd (cm) | 0.025 | 0.895 | 0.051 | 0.787 | 0.050 | 0.898 | 0.283 | 0.130 | 0.246 | 0.191 | 0.094 | 0.620 |
| LVSd (cm) | -0.055 | 0.774 | 0.007 | 0.969 | 0.226 | 0.559 | 0.304 | 0.102 | 0.159 | 0.402 | 0.018 | 0.926 |
| LVPWd (cm) | 0.415 | **0.022** | 0.515 | **0.004** | -0.370 | 0.327 | 0.268 | 0.153 | 0.237 | 0.208 | 0.218 | 0.247 |
| IVSd z-score | -0.006 | 0.973 | -0.010 | 0.956 | -0.435 | 0.242 | -0.103 | 0.586 | -0.232 | 0.218 | -0.188 | 0.319 |
| LVDd z-score | -0.156 | 0.409 | -0.119 | 0.533 | 0.333 | 0.381 | 0.167 | 0.378 | -0.010 | 0.960 | -0.032 | 0.866 |
| LVSd z-score | -0.309 | 0.097 | -0.258 | 0.168 | 0.333 | 0.381 | 0.087 | 0.646 | -0.115 | 0.543 | -0.141 | 0.458 |
| LVPWd z-score | 0.212 | 0.262 | 0.308 | 0.098 | -0.133 | 0.732 | 0.105 | 0.579 | 0.055 | 0.773 | 0.127 | 0.502 |
| FS (%) | -0.167 | 0.378 | -0.120 | 0.527 | -0.145 | 0.709 | -0.046 | 0.810 | -0.137 | 0.469 | 0.031 | 0.873 |
| EF (%) | -0.077 | 0.685 | -0.040 | 0.836 | -0.068 | 0.862 | -0.009 | 0.960 | -0.147 | 0.439 | -0.055 | 0.773 |
| LA (cm) | 0.093 | 0.626 | 0.228 | 0.226 | 0.883 | **0.002** | 0.250 | 0.182 | 0.127 | 0.503 | 0.181 | 0.337 |
| LA z-score | 0.009 | 0.962 | 0.166 | 0.380 | 0.833 | **0.005** | 0.135 | 0.478 | -0.015 | 0.938 | 0.114 | 0.548 |
| Aortic root (cm) | 0.415 | 0.023 | 0.388 | **0.034** | -0.483 | 0.187 | 0.359 | 0.051 | 0.164 | 0.388 | -0.096 | 0.613 |
| Aortic root z-score | 0.159 | 0.402 | 0.154 | 0.417 | -0.450 | 0.224 | 0.102 | 0.592 | -0.025 | 0.895 | -0.156 | 0.412 |
| Mitral E | -0.372 | **0.043** | -0.397 | **0.030** | 0.561 | 0.116 | -0.230 | 0.221 | -0.181 | 0.337 | -0.011 | 0.954 |
| Mitral A | -0.136 | 0.473 | -0.170 | 0.369 | 0.083 | 0.831 | 0.009 | 0.962 | 0.151 | 0.426 | 0.248 | 0.186 |
| DT | 0.102 | 0.590 | -0.091 | 0.631 | -0.594 | 0.092 | -0.457 | **0.011** | -0.201 | 0.287 | -0.307 | 0.099 |
| Mitral E z-score | -0.356 | 0.053 | -0.400 | **0.029** | 0.550 | 0.125 | -0.234 | 0.213 | -0.182 | 0.336 | -0.013 | 0.945 |
| Mitral A z-score | -0.126 | 0.507 | -0.160 | 0.398 | 0.083 | 0.831 | 0.014 | 0.942 | 0.152 | 0.423 | 0.238 | 0.206 |
| DT z-score | 0.051 | 0.790 | -0.132 | 0.488 | -0.617 | 0.077 | -0.492 | **0.006** | -0.251 | 0.182 | -0.320 | 0.084 |
| IVRT | 0.262 | 0.162 | 0.068 | 0.721 | 0.211 | 0.586 | -0.027 | 0.886 | -0.054 | 0.778 | -0.228 | 0.226 |
| Mitral E/A | -0.035 | 0.853 | -0.053 | 0.781 | -0.017 | 0.966 | -0.137 | 0.471 | -0.246 | 0.191 | -0.298 | 0.110 |
| Mitral E/A z-score | -0.018 | 0.926 | -0.040 | 0.833 | 0.050 | 0.898 | -0.174 | 0.357 | -0.235 | 0.212 | -0.261 | 0.164 |
| Aortic velocity | -0.373 | **0.043** | -0.232 | 0.217 | 0.617 | 0.077 | 0.087 | 0.647 | -0.014 | 0.943 | 0.188 | 0.321 |
| PA velocity | -0.243 | 0.196 | -0.176 | 0.353 | 0.377 | 0.318 | 0.216 | 0.251 | 0.169 | 0.372 | 0.244 | 0.195 |
| Descending Ao velocity | -0.418 | **0.022** | -0.266 | 0.155 | 0.717 | **0.030** | -0.131 | 0.492 | -0.111 | 0.558 | 0.096 | 0.612 |
| **Tissue Doppler Parameters** |  |  |  |  |  |  |  |  |  |  |  |  |
| S′ | -0.090 | 0.636 | -0.050 | 0.795 | -0.067 | 0.865 | 0.132 | 0.488 | 0.031 | 0.871 | 0.015 | 0.937 |
| E′ | -0.244 | 0.194 | -0.186 | 0.326 | 0.300 | 0.433 | 0.010 | 0.960 | -0.169 | 0.373 | -0.018 | 0.923 |
| A′ | -0.128 | 0.499 | -0.003 | 0.986 | 0.317 | 0.406 | 0.187 | 0.322 | 0.187 | 0.323 | 0.314 | 0.091 |
| E′/A′ | -0.057 | 0.765 | -0.146 | 0.440 | 0.176 | 0.651 | -0.196 | 0.299 | -0.431 | **0.017** | -0.456 | **0.011** |
| S′ z-score | -0.154 | 0.417 | -0.092 | 0.627 | 0.017 | 0.966 | 0.089 | 0.638 | 0.016 | 0.932 | 0.060 | 0.753 |
| E′ z-score | -0.262 | 0.161 | -0.198 | 0.294 | 0.333 | 0.381 | 0.011 | 0.954 | -0.181 | 0.338 | -0.027 | 0.888 |
| A′ z-score | -0.134 | 0.479 | -0.005 | 0.980 | 0.317 | 0.406 | 0.188 | 0.319 | 0.185 | 0.328 | 0.313 | 0.092 |
| E′/A′ z-score | -0.103 | 0.589 | -0.184 | 0.331 | 0.251 | 0.515 | -0.224 | 0.233 | -0.447 | **0.013** | -0.453 | **0.012** |
| MPI | 0.136 | 0.473 | 0.063 | 0.741 | 0.153 | 0.694 | 0.112 | 0.557 | 0.062 | 0.746 | 0.053 | 0.781 |
| E/E′ | 0.024 | 0.900 | -0.065 | 0.731 | -0.218 | 0.574 | -0.133 | 0.482 | 0.078 | 0.682 | 0.005 | 0.979 |
| MAPSE | 0.239 | 0.220 | 0.137 | 0.486 | -0.216 | 0.608 | -0.042 | 0.830 | 0.025 | 0.901 | -0.011 | 0.957 |
| **Longitudinal Strain and  Time-to-Peak Parameters** |  |  |  |  |  |  |  |  |  |  |  |  |
| LV EDV (bi-plane) | 0.300 | 0.121 | 0.316 | 0.101 | -0.619 | 0.102 | 0.453 | **0.015** | 0.286 | 0.141 | 0.112 | 0.570 |
| LV ESV (bi-plane) | 0.309 | 0.110 | 0.300 | 0.121 | -0.381 | 0.352 | 0.434 | **0.021** | 0.268 | 0.168 | 0.152 | 0.439 |
| LV EF (bi-plane) | -0.154 | 0.433 | -0.136 | 0.491 | -0.024 | 0.955 | -0.205 | 0.295 | -0.136 | 0.490 | -0.167 | 0.397 |
| LV AP2 peak L time | 0.104 | 0.585 | 0.099 | 0.601 | -0.383 | 0.308 | 0.260 | 0.165 | 0.376 | **0.041** | 0.357 | 0.053 |
| LV AP3 peak L time | 0.283 | 0.129 | 0.251 | 0.181 | -0.017 | 0.966 | 0.296 | 0.112 | 0.371 | **0.043** | 0.218 | 0.248 |
| LV AP4 peak L time | 0.372 | 0.043 | 0.346 | 0.061 | -0.183 | 0.637 | 0.246 | 0.190 | 0.365 | **0.047** | 0.260 | 0.165 |
| LV global peak L time | 0.081 | 0.671 | 0.079 | 0.677 | 0.467 | 0.205 | 0.216 | 0.251 | 0.260 | 0.165 | 0.276 | 0.140 |
| LV AP2 peak L strain | 0.140 | 0.461 | 0.119 | 0.530 | -0.469 | 0.203 | 0.242 | 0.198 | 0.287 | 0.124 | 0.256 | 0.171 |
| LV AP3 peak L strain | 0.144 | 0.448 | 0.178 | 0.346 | 0.583 | 0.099 | 0.009 | 0.963 | 0.346 | 0.061 | 0.509 | **0.004** |
| LV AP4 peak L strain | 0.185 | 0.328 | 0.192 | 0.310 | -0.633 | 0.067 | 0.241 | 0.199 | 0.454 | **0.012** | 0.410 | **0.025** |
| LV global peak L strain | 0.205 | 0.278 | 0.173 | 0.360 | -0.150 | 0.700 | 0.193 | 0.306 | 0.437 | **0.016** | 0.456 | **0.011** |
| **Circumferential Strain  and Time-to-Peak Parameters** |  |  |  |  |  |  |  |  |  |  |  |  |
| LV SAX A peak C time | 0.045 | 0.814 | 0.067 | 0.725 | 0.000 | 1.000 | 0.161 | 0.396 | 0.511 | **0.004** | 0.475 | **0.008** |
| LV SAX M peak C time | 0.096 | 0.613 | 0.147 | 0.438 | 0.033 | 0.932 | 0.242 | 0.197 | 0.255 | 0.174 | 0.180 | 0.342 |
| LV SAX B peak C time | 0.299 | 0.109 | 0.264 | 0.159 | 0.017 | 0.966 | 0.563 | **0.001** | 0.510 | **0.004** | 0.314 | 0.091 |
| LV global peak C time | -0.133 | 0.490 | -0.165 | 0.392 | -0.267 | 0.488 | 0.116 | 0.550 | 0.185 | 0.336 | 0.042 | 0.827 |
| LV SAX A peak C strain | -0.140 | 0.461 | -0.103 | 0.588 | 0.217 | 0.576 | 0.100 | 0.598 | 0.245 | 0.191 | 0.363 | **0.049** |
| LV SAX M peak C strain | -0.022 | 0.910 | 0.072 | 0.704 | -0.133 | 0.732 | 0.420 | **0.021** | 0.318 | 0.087 | 0.222 | 0.239 |
| LV SAX B peak C strain | 0.354 | 0.055 | 0.323 | 0.082 | 0.050 | 0.898 | 0.428 | **0.018** | 0.360 | 0.051 | 0.254 | 0.175 |
| LV global peak C strain | 0.057 | 0.770 | 0.144 | 0.455 | -0.033 | 0.932 | 0.406 | **0.029** | 0.425 | **0.022** | 0.357 | 0.057 |
| **ACMQ Parameters** |  |  |  |  |  |  |  |  |  |  |  |  |
| LV global endo L peak ACMQ | 0.200 | 0.290 | 0.163 | 0.389 | -0.150 | 0.700 | 0.185 | 0.328 | 0.433 | **0.017** | 0.452 | **0.012** |
| LV AP4 endo L peak ACMQ | 0.254 | 0.176 | 0.221 | 0.240 | -0.650 | 0.058 | 0.239 | 0.204 | 0.462 | **0.010** | 0.380 | **0.038** |
| LV AP2 endo L peak ACMQ | 0.174 | 0.358 | 0.077 | 0.685 | -0.417 | 0.265 | 0.206 | 0.274 | 0.265 | 0.157 | 0.198 | 0.295 |
| LV AP3 endo L peak ACMQ | 0.134 | 0.480 | 0.130 | 0.495 | 0.550 | 0.125 | -0.021 | 0.912 | 0.321 | 0.084 | 0.470 | **0.009** |
| LV global endo C peak ACMQ | -0.039 | 0.849 | -0.013 | 0.948 | 0.033 | 0.932 | 0.243 | 0.231 | 0.270 | 0.181 | 0.223 | 0.273 |
| LV SAX B endo C peak ACMQ | 0.263 | 0.185 | 0.210 | 0.293 | 0.100 | 0.798 | 0.382 | 0.050 | 0.307 | 0.119 | 0.251 | 0.207 |
| LV SAX M endo C peak ACMQ | -0.255 | 0.209 | -0.185 | 0.365 | -0.133 | 0.732 | 0.278 | 0.168 | 0.162 | 0.430 | 0.130 | 0.527 |
| LV SAX A endo C peak ACMQ | -0.131 | 0.500 | -0.105 | 0.588 | 0.267 | 0.488 | 0.101 | 0.601 | 0.340 | 0.071 | 0.449 | **0.014** |
| **Area and Mass Parameters** |  |  |  |  |  |  |  |  |  |  |  |  |
| LV EDA | 0.332 | 0.105 | 0.250 | 0.228 | -0.133 | 0.732 | 0.265 | 0.200 | 0.508 | **0.009** | 0.275 | 0.183 |
| LV ESA | 0.135 | 0.519 | 0.081 | 0.701 | -0.117 | 0.765 | 0.356 | 0.081 | 0.419 | **0.037** | 0.221 | 0.289 |
| LV FAC (%) | 0.421 | **0.036** | 0.346 | 0.090 | 0.133 | 0.732 | -0.106 | 0.613 | 0.023 | 0.913 | -0.030 | 0.885 |
| LV Mass (cubed), g | 0.270 | 0.148 | 0.314 | 0.092 | -0.450 | 0.224 | 0.345 | 0.062 | 0.305 | 0.102 | 0.184 | 0.331 |

*Spearman correlation analysis was used. r: correlation coefficient. Trp T: troponin T; CK-MB: creatine kinase-MB; BNP: brain natriuretic peptide; CRP: C-reactive protein; NLR: neutrophil-to-lymphocyte ratio; SII: systemic immune-inflammation index; IVSd: interventricular septal thickness in diastole; LVDd: left ventricular end-diastolic diameter; LVSd: left ventricular end-systolic diameter; LVPWd: left ventricular posterior wall thickness in diastole; FS: fractional shortening; EF: ejection fraction; LA: left atrium; DT: deceleration time; IVRT: isovolumic relaxation time; E: early diastolic transmitral flow velocity; A: late diastolic transmitral flow velocity; E′: early diastolic mitral annular velocity; A′: late diastolic mitral annular velocity; MPI: myocardial performance index; MAPSE: mitral annular plane systolic excursion; EDV: end-diastolic volume; ESV: end-systolic volume; AP2: apical 2-chamber view; AP3: apical 3-chamber view; AP4: apical 4-chamber view; SAX: short-axis; L: longitudinal; C: circumferential; ACMQ: area change myocardial quotient; EDA: end-diastolic area; ESA: end-systolic area; FAC: fractional area change; Ao: aortic; PA: pulmonary artery.*
